# Corneal dose response from exposure to a *Q*-switched laser at a central wavelength of 1645 nm using a rabbit model

**DOI:** 10.1117/1.JBO.31.5.054703

**Published:** 2026-02-11

**Authors:** Joseph J. Chue-Sang, Xomalin G. Peralta, Joseph E. Clary, Andrea Smith, Amanda Peterson, Matthew E. Macasadia, Amanda J. Tijerina, Gary D. Noojin, Maximillian V. Hart, Charles F. Schwarten, Wesley T. Kinerk, Lyndsey M. Ferris, Emily N. Boice

**Affiliations:** a SAIC, Joint Base San Antonio, Fort Sam Houston, Texas, United States; b Conceptual MindWorks, Inc., Joint Base San Antonio, Fort Sam Houston, Texas, United States; c Air Force Research Laboratory, Joint Base San Antonio, Fort Sam Houston, Texas, United States

**Keywords:** laser, safety, wavelength, nanosecond, ocular, eye, cornea, damage threshold, optical coherence tomography

## Abstract

**Significance:**

Laser safety studies of the eye are well documented for visible wavelength and continuous wave lasers. There are fewer experimental results for infrared wavelengths and pulsed lasers.

**Aim:**

We aim to fill the gap at 1645 nm for single nanosecond pulse duration exposures of rabbit cornea and determine the threshold radiant exposure to generate lesions 50% of the time (estimated dose ${\mathrm{ED}}_{50}$).

**Approach:**

Images of the cornea during exposures were acquired using slit lamp microscopy and optical coherence tomography. A histological analysis helped provide dosimetry relationships with morphology and mechanisms of the damage.

**Results:**

We measured the energy ${\mathrm{ED}}_{50}$ value at 3.86±0.085  mJ utilizing the slit lamp biomicroscopy. Incorporating the experimental spot size diameter, this corresponds to a peak radiant exposure of $102\;J/{\mathrm{cm}}^{2}$. By contrast, the average radiant exposure ${\mathrm{ED}}_{50}$ over a 1-mm diameter limiting aperture as per the ANSI Z.136 convention was $0.49\;J/{\mathrm{cm}}^{2}$. Additional analysis via optical coherence tomography (OCT) and histology examined the severity and degree of damage.

**Conclusion:**

This experimental approach performed well to characterize damage and identify damage thresholds to inform the laser safety standard community of the accuracy of current exposure limits.

## 1 Introduction

The American National Safety Institute (ANSI) laser safety standards (ANSI Z136.1)^1^ include maximum permissible exposure (MPE) limits categorized by type of laser source, wavelength, and exposure duration. The MPEs for ocular laser exposures shorter than 10 s are commonly expressed as radiant exposure and use a limiting aperture for calculations of the MPE. In particular, the limiting aperture for corneal exposures is defined as a circle with a diameter of 1 mm. Currently, lasers with a wavelength of 1645 nm and a pulse duration in the nanosecond ($1\;\mathrm{ns}=1\;\mathrm{ns}=10^{-9}\;s$) regime are used in rangefinding, tracking, and material manufacturing for both civilian and military applications. The current point-source MPE value listed in the ANSI Z136.1 with these specifications is $1\;J/{\mathrm{cm}}^{2}$, indicating exposures up to $1\;J/{\mathrm{cm}}^{2}$ would be considered safe. However, this part of the spectrum has no biological data supporting the standard, and more information is required to clarify their safe use in applications that would cause human exposures. MPE values with limited or no biological data within a spectral region are usually interpolated with values of water absorption coefficients and thresholds at shorter wavelengths.^2^^,^^3^ The wavelengths closest to 1645 nm, where laser-induced cornea damage has been reported, are 1540, 1550, 1573, and 1732 nm.^2^^,^^4^ Understanding the relationship between laser exposure conditions and the resulting bioeffects is called dosimetry, which is used to determine damage thresholds that provide a framework for ANSI subcommittees when creating or modifying laser safety guidelines. The Air Force Research Laboratory (AFRL) has been investigating and publishing laser damage thresholds for over 40 years to inform Department of Defense policies (AFI 48-139,^5^ DoDI 6055.15),^6^ national,^1^ and international (STANAG 3606, IEC 80625)^7^ laser safety standards. Currently, one question AFRL has been addressing is the scenario where the exposure laser beam has a significantly smaller diameter than the limiting aperture.

The human visual system uses visible light wavelengths (400 to 700 nm) to form and process images. The eye serves the initial function of the system, which is a neural tissue considered a peripheral extension of the central nervous system. The complex multilayered retina performs the conversion of photons to neural signals and transmits those signals to the visual cortex via the lateral geniculate nucleus, where higher-order processing of visual information is achieved.

The ocular functions begin with the anterior-most tissue called the cornea. The cornea focuses light onto the retina at the posterior portion of the eye. The lens performs some focusing of light but is primarily used in near-field accommodation. With the focal length of the cornea and lens system, and chromatic aberrations in ocular tissue, light in the range of 555 to 579 nm is best focused on the retina. Light in the infrared (IR) spectrum will focus beyond the retina into the choroidal structures. In addition, the cornea provides a barrier of protection against external threats such as pathogens, foreign bodies, ultraviolet irradiation, and physical injury and is thus susceptible to injury. Depending upon the wavelength, directed electromagnetic energy may also cause injury to the cornea.

Corneas are also covered in a thin layer of water, mucous, and oil known as the tear film. This wetting agent is secreted by the lacrimal glands and ocular conjunctiva and is applied over the cornea when the eyelids blink. As water is generally an absorber of NIR wavelength light, the tear layer provides some protection against the directed energy of these wavelengths.

Human corneas consist of five layers: beginning at the outermost (anterior) layer, they are the epithelium, Bowman’s layer, stroma, Descemet’s membrane, and the endothelium. Some of these layers are not present in all animal species. Although porcine and rabbit corneas do not have Bowman’s membrane,^8^ rabbits have become a widely accepted model for measuring corneal damage thresholds.^9^^–^^15^ Conversely, swine are being explored as an animal model for replacing nonhuman primates (NHPs) for measuring retinal laser damage thresholds due to ethical concerns and limited resources.^16^ Although swine have yet to be validated and accepted as the preferred animal model, they are more readily available and can provide valuable information and trends for retinal damage thresholds.

Experiments have identified photochemical, photothermal, and photomechanical processes as possible injury mechanisms from laser exposure. Laser variables influencing the damage mechanisms include energy content, the repetition rate of the laser, and wavelength.^17^^,^^18^ Lasers operate at repetition rates ranging from continuous wave (CW) to pulsed lasers with pulse durations commonly ranging from microseconds to femtoseconds, depending on the application. CW lasers are generally linked to causing thermal injuries, whereas pulsed lasers are thought to scale toward ablation and more mechanical–based injuries as pulse duration shortens. Ablation is the ejection of tissue such as vaporization or plasma generation caused by heat deposition.^17^ The addition of mechanical force to a tissue caused by shorter pulsed lasers may lower the thermal requirements for damage, resulting in an injury atypical of a completely thermal injury as produced by a CW laser. Ultraviolet (UV) wavelengths can cause photochemical injury by alterations of certain biomolecules, particularly within the UV-B (280 to 315 nm) range.^17^^,^^19^ When absorbed in the cornea at sufficient dosage, UV exposure can destroy the epithelial cell layer. Further exposure can extend the damage through the layers of the cornea. The NIR wavelengths similarly prioritize causing injury in the cornea due to its optical properties. This indicates UV and NIR absorption in the corneal tissue would protect the retina from UV [<380  nm] and IR [1400 nm to 1 mm] wavelengths, which are both invisible to the human eye.^20^^,^^21^

**Fig. 1:**
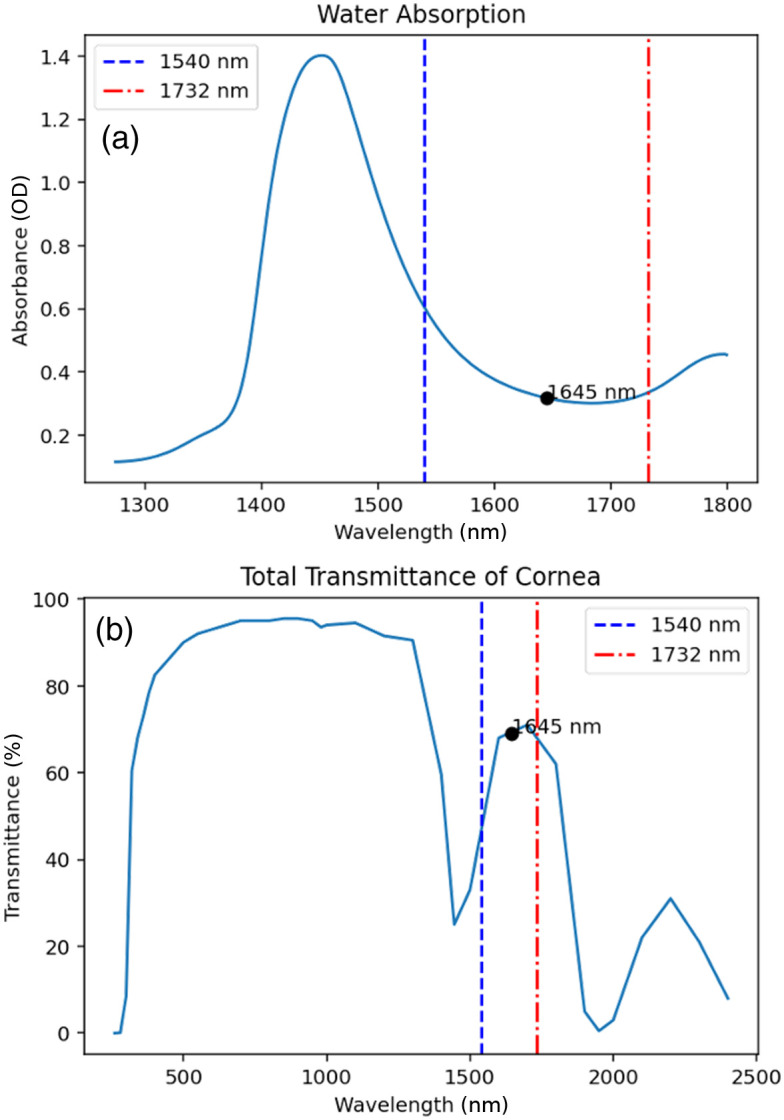
(a) Transmittance of the cornea as a function of wavelength measured using nonhuman primate (NHP) eyes by Boettner et al.^21^ Light with a wavelength of 1645 nm has a transmittance greater than 60%, (b) water absorption spectrum.^22^ The dashed lines indicate where experimental data exist for particular wavelengths (1540, blue; 1732, red). The wavelength used in this study, 1645 nm, is at a local minimum for water absorption, which is the major attenuator. No experimental data exists on corneal laser exposure damage at this wavelength.

Although the cornea is expected to be injured from a NIR laser exposure, several factors suggest the need for a preliminary study to determine if the cornea or retina was a target, which is most susceptible at 1645 nm. The cornea has high transmittance and relatively low water absorption at 1645 nm compared with the surrounding wavelengths of 1540 and 1732 nm, where laser damage thresholds exist in the literature (see Fig. 1). This relative minimum in absorbance at 1645 nm supports the hypothesis that light at this wavelength could transmit through the aqueous and vitreous humors with sufficient energy to injure the retina. To test this hypothesis, a pilot study^23^ was previously conducted to determine which ocular tissue had the lowest damage threshold when exposed to an ns laser at 1645 nm.^23^

Whereas a rabbit model is preferred for corneal laser bioeffects, this early hypothesis suggested the possibility of retinal damage, where a porcine model is the preferred animal model. The Yucatan minipig (*Sus scrofa domesticus*) has been utilized for previous laser exposure studies.^16^^,^^24^^,^^25^ The eyes of swine are similar to humans in terms of dimensions, physiology, and optical properties.^26^^–^^29^ Although the porcine retina lacks a true fovea and macula, which are found in the human eye, it does have a macular streak exhibiting a heightened density of cone photoreceptor cells.^30^^,^^31^ It also lacks a tapetum lucidum, a reflective layer of the choroid that reflects light back to the retina to improve night vision, which is also not present in humans. The porcine retina is also holangiotic, or fully vascularized, similar to human and NHP anatomies.^32^^–^^37^ The branching vasculature throughout the porcine retina provides guidance in the placement and organization of laser exposure sites and can be viewed using standard human ophthalmic devices, such as a fundus camera. The similarities between human and porcine ocular anatomy have led to the development of numerous porcine models of ocular injury and disease in recent years.^38^^–^^44^

In the prior study, porcine eyes were exposed to different energies and numbers of pulses. In compliance with modern practice for establishing exposure guidelines, the eyes were examined for corneal damage at 1 h and retinal damage at 24 h postexposure. The laser optical system was designed to produce a collimated beam to take advantage of the subject’s cornea and lens to focus light. The eye’s ability to focus light is influenced by its component shapes and the wavelength of the light it refracts and is optimized for visible light. The longer wavelength investigated in this study would be focused behind the retina. However, this is preferable to sending a focused beam to the cornea, which would diverge to be completely out of focus by the time it reached the retina. The system utilized in this pilot study had a large final beam size (∼1  mm) to avoid damaging the laser exposure system’s optical components.^23^

Lesions can have differences in chemical and morphological characteristics that may be tissue-specific. Corneal lesions typically appear as opaque spots in otherwise transparent and healthy tissue. Superficial lesions, which mainly affect the epithelium and can heal without medical assistance, are expected to appear as white opacities. Retinal lesions can be more difficult to distinguish due to the retina’s natural pigmentation and striations, which become more pronounced in older swine, hence the preference for juvenile animals for ocular studies. The variegated appearance of the retina makes comparison with baseline images with laser-induced alterations imperative. By contrast, imperfections in the cornea are commonly associated with dryness, which, when severe, can confound lesion confirmation. The pilot study was not concerned with finding a statistically significant damage threshold value but rather a radiant exposure window where damage occurred in the cornea or retina. The cornea was altered at a lower energy than the retina when exposed to a 77-ns pulse at 1645 nm. The peak radiant exposure per pulse was $3.58\;J/{\mathrm{cm}}^{2}$ using a beam diameter (${1/e}^{2}$) of 1.01±0.06  mm. Note that while the ${1/e}^{2}$ beam was measured experimentally and is used to calculate average radiant exposure, it is the 1/e beam diameter that is used to calculate peak radiant exposure. Dividing the ${1/e}^{2}$ beam diameter by the square root of 2 approximates the 1/e beam diameter. The damage threshold for the porcine cornea occurred between the 11th and 15th pulses, for a total delivered peak radiant exposure range of 39.5 to $53.7\;J/{\mathrm{cm}}^{2}$.

The results of this pilot study informed the design of the study described here to minimize the use of laboratory animals, as per the Department of Defense Instruction 3216.1. The goal of the current study was to provide laser bioeffects parameterized data to the laser safety community for corneal exposures to 77-ns pulses at 1645 nm. This is calculated with a single pulse Probit estimated dose for 50% probability of damage (${\mathrm{ED}}_{50}$) for minimum visible lesions (MVL) or greater in the rabbit cornea.

## 2 Methods

Five female Dutch-belted rabbits (*Oryctolagus cuniculus*) were used to assess the corneal MVL threshold. This number was chosen to ensure biological variability. All procedures were performed in accordance with an Institutional Animal Care and Use Committee (IACUC)-approved Animal Use Protocol in an AAALAC-accredited facility with established animal welfare standards, compliant with DoD Instruction 3216.01^45^; U.S. Department of Agriculture Animal Welfare Act and Regulations^46^; and Defense Health Agency Multi-Service Regulation (DHA-MSR) 6025.02,^47^ and *The Guide for the Care and Use of Laboratory Animals*, 8th Edition, National Research Council (2011).^48^ The AFRL at Joint Base San Antonio (JBSA), Fort Sam Houston, Texas, has been accredited by AAALAC International since 1967.

Subjects were anesthetized and maintained under anesthesia for the duration of the eye exams and laser exposure procedures. Peribulbar lidocaine (Phoenix) injections were performed, and scleral stay sutures were placed to limit eye movement. The eyelashes were clipped if their length could interfere with the experiment, and the pupils were dilated using a 1% tropicamide/10% phenylephrine solution (Akorn). The eyes were kept lubricated using GenTeal (Alcon) or another ophthalmic ointment when not in use. Baseline imaging of the cornea was completed prior to exposure to assess the cornea’s health and identify locations for laser targeting. The subject was then moved to the laser laboratory for the corneal laser exposures. Proparacaine (0.5%) eye drops (Akorn) were administered for pain management before laser exposures. After completion of laser exposures, lubricating ointment was reapplied to the eye, the subject’s vitals were monitored, and the animal was evaluated during the 1-h postexposure examination.

The anesthetized subject was placed in sternal recumbency in an adjustable five-axis positional animal stage. The stage was designed to manipulate the placement of the eye while the subject was wrapped with a warming blanket during laser exposures and imaging. The eye designated for exposure was kept open by inserting a wire speculum. The subject’s eye could also be held open by hand as needed during imaging. To prevent the eyes from drying out when the speculum was in position, they were irrigated with 0.9% normal saline every 20 to 30 s.

A slit lamp biomicroscope (Haag-Streit, BX-900) and optical coherence tomography (OCT) (Heidelberg Engineering, Spectralis HRA + OCT w/anterior segment attachment) were used for optical examinations. The subject was positioned on the stage, and the surface of the cornea was maneuvered to within the instrument’s working distance. These ophthalmic instruments were designed for human patients and were modified for experimental use with animals, whose eyes are located more laterally.

The slit lamp biomicroscope recorded baseline and postexposure images of the cornea. Corneal epithelial abnormalities were highlighted by applying fluorescein (GKPA Ophthalmic Fluoro Touch Strips) to the cornea. Eyes were examined and imaged 1 h after laser exposures. Depending on scheduling, some eyes were re-examined 24 and 72 h after laser exposures. Several eyes were collected postmortem for histological analysis postexposure. Probit^49^ regression determined the effective radiant exposure with a 50% probability of causing a lesion (${\mathrm{ED}}_{50}$). This value was compared with the current point-source MPE in the ANSI Z136.1 for a laser at 1645 nm with pulse durations within the ns regime.

### 2.1 Exposure Setup

The experiment used an ELPN-1645 (IPG, 1645±5  nm) fiber-pumped Er:YAG ns laser, which is specified to produce a 20-mJ pulse at a 1-kHz repetition rate. The Q-switch system had a nominal pulse width of 77 ns during the experiment. To determine the threshold ${\mathrm{ED}}_{50}$ dose on the cornea, the optical layout produced a converging beam focused on the cornea. Minimizing the beam size increased the delivered laser irradiance to levels that generate damage from single pulses. Considering the maximum output of the laser, expected losses from the optical layout, and the results of a pilot study,^23^ a laser beam diameter of around 100  μm was chosen.

**Fig. 2:**
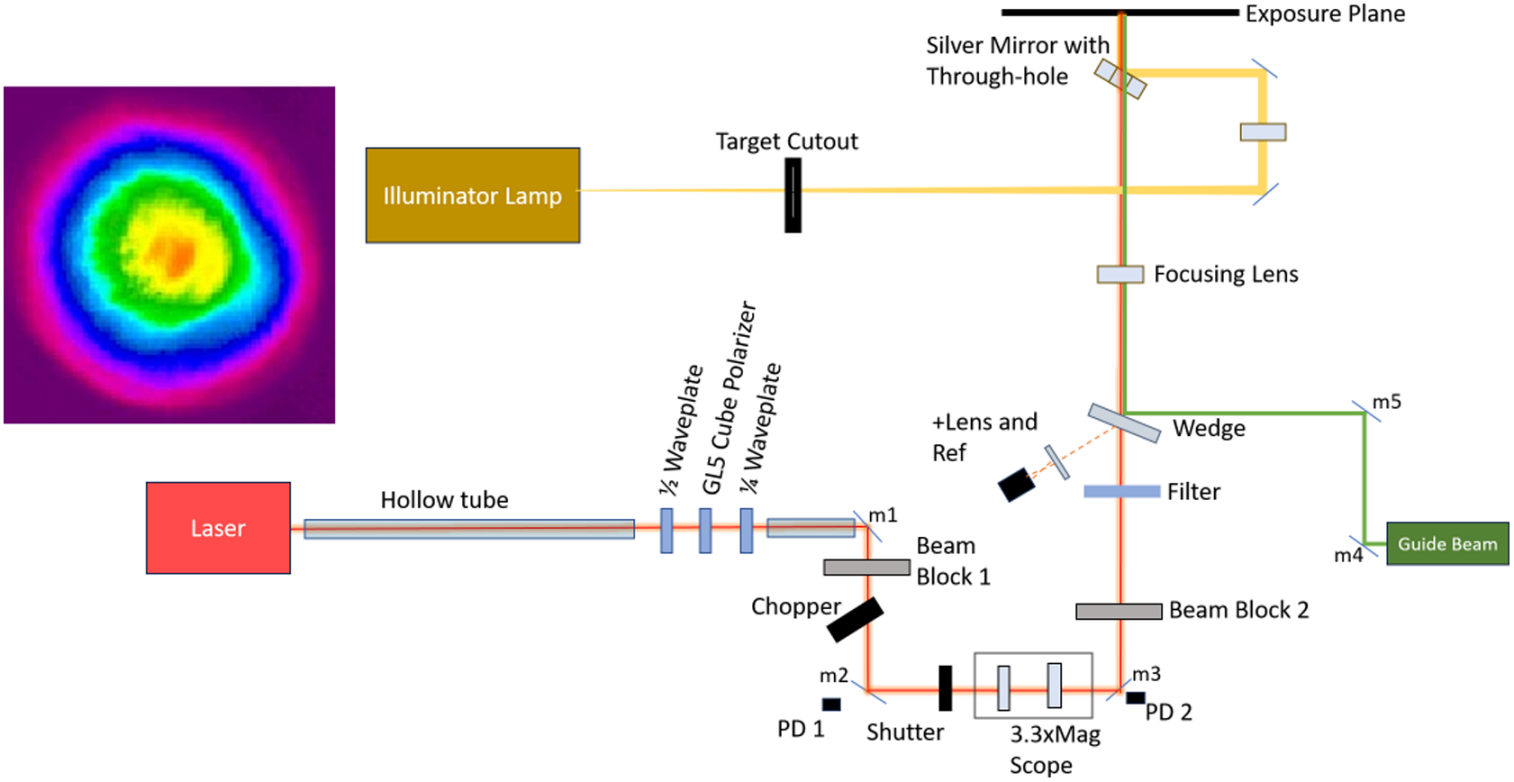
Optical layout and resultant beam profile. The beam profile was imaged after allowing the beam to expand past the exposure plane to avoid damaging the camera. Due to the hazard of back-reflections returning to and damaging the laser, the first leg of the layout was caged around an optical isolator. The tubing and optical isolator (polarizer/waveplate combination) at the beginning of the beam path were implemented to prevent back reflections from damaging the laser. The beam’s repetition rate was reduced from 1000 to 100 Hz using the chopper. The beam was then cut to a single pulse exposure using the shutter before being expanded by a two-lens telescope. ND filters and the quarter-waveplate in the optical isolator were used to adjust the energy delivered. The beam was then focused through a hole in the final mirror onto the cornea. The final mirror was used to project a reticle onto the cornea to aid exposure targeting.

Figure 2 shows the optical layout of the laser system. The initial output of the system was a beam with a 1-mm diameter, providing a radiant exposure that exceeded the damage threshold of the dielectric mirrors when the laser was running at maximum energy output. Therefore, a long path length (∼1  m on the first leg) was incorporated into the optical exposure setup to allow the beam to naturally diverge at its native 1 mrad divergence. This first leg passed through an optical isolator that prevented laser back reflections from entering the laser cavity and damaging the device. The isolator used a half-wave plate (AHWP05M-1600, Thorlabs), cube polarizer (GT5, Thorlabs), and quarter-wave plate (AQWP05M-1600, Thorlabs) at the cost of energy output. The 1 kHz repetition rate of the laser was reduced to 100 Hz using a custom-made 20/2 chopper wheel. A photodiode (PDA10CF, Thorlabs, Fig. 2 PD1) read the new frequency of the laser pulses impeded and reflected from the shutter (VS25, Uniblitz), and a data acquisition card (National Instruments) logged the photodiode output. A custom LabVIEW program used the 100 Hz pulse train to synchronize a 9.8-ms length shutter window output. Once an event was triggered, the program used the next pulse seen as time zero and delayed the shutter output transistor-transistor logic (TTL) pulse by 2.5 ms so that the shutter isolated a single pulse from the pulse train. A delay generator (DG645, Stanford) also used this TTL shutter pulse as a zero-point and added a delay for external trigger synchronization of the measurement devices further down the optical layout. A second detector (Fig. 2 PD2) located after the shutter and telescope confirmed single pulse exposures by measuring leakage from a turning mirror (DSMP1180L, Thorlabs) and captured the pulse on an oscilloscope (3054B, Tektronix).

Further down the path, the appropriate filters in conjunction with the quarter-wave plate in the optical isolator at the beginning of the layout attenuated the energy of the laser beam. This combination provided the range of radiant exposures needed to complete a Probit analysis of the MVL threshold dosage. A fused silica reference window (Fig. 2 Wedge), which reflected 2% of the incoming beam into a low-energy detector (J10-MB-LE, Coherent), estimated the delivered energy. This reference detector and meter (LabMax-Top, Coherent) allowed the user to estimate the delivered energy based on a ratio between the reference energy and exposure energy, which was calibrated before and after performing exposures.

The telescope located after the shutter and the lens located at the end of the optical layout conditioned the beam. The telescope expanded the beam by 3.3 times and collimated it using two lenses (LC4252-C, LA4380-C, Thorlabs). The final focusing lens (LA4579-C, Thorlabs) had a focal length of 300 mm and produced a final beam waist diameter (${1/e}^{2}$) of 98.25±3.42  μm at the exposure plane. The radiant exposure measurements incorporated the small day-to-day changes of the spot size due to laser variability.

**Fig. 3:**
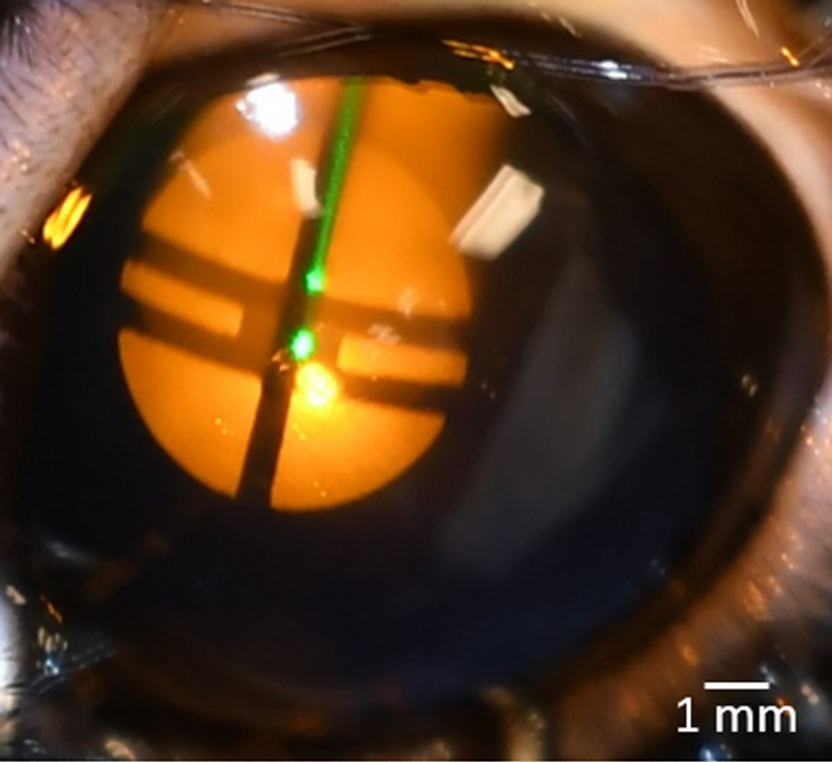
Example of target illuminating the eye of a Dutch-belted rabbit with a green HeNe laser centered in the crosshairs and collinear with the exposing laser. The second green spot located above the center is a reflection. A suture can be seen in the top-right. The target diameter was ∼5  mm. Keeping the target image in focus meant the cornea was at the beam waist [exposure plane] of the laser. A live video of this view was captured and displayed for staff to help determine a good exposure site devoid of imperfections such as dryness or previous exposures as the animal was maneuvered on the five-axis stage.

Projecting a target onto the cornea enabled precise placement of laser exposures and discerning the focal depth of the exposure plane (see Fig. 3). The illuminator functioned by launching light from a fiber-coupled light-emitting diode (LED) (M590F3, Thorlabs) into a 1-m-long optical fiber (M35L02, Thorlabs). A 40-mm lens (AL5040M, Thorlabs) projected an image of the output fiber tip to infinity to achieve collimation before it passed through a masking material with a 25.4-mm diameter crosshairs pattern cutout. The collimated target image then traveled for 1500 mm after the target before reaching a 300-mm lens (AC254-300-A-ML, Thorlabs) that focused the target image on the exposure plane of the laser system. The distances between optical components and focal distances of the lenses were set so that the target would have a diameter of ∼5  mm on the cornea (0.2 magnification), and the focus of the target would be sharpest at the proper position of the laser exposure plane. Moving away from the beam waist would blur the target image and inform the operator that the subject’s positioning needed to be adjusted. The final 50 mm mirror after the 300 mm lens that reflected the target onto the eye had an ∼6  mm (1/4″) diameter hole drilled at a 45-deg angle through its center to allow the laser to pass through while still reflecting the target image 90 deg toward the exposure plane. Alignment of the illumination with the hole in the final mirror was critical to ensure the laser exposure was centered on the target on the eye. A green HeNe laser (LDS5, Thorlabs) was also set collinear with the exposure laser that was center to the target crosshairs as further aiming assistance.

A DSLR camera (Nikon, D7500) mounted above the final mirror (with a hole) captured the event before and after every exposure at 20.9 megapixels resolution and was invaluable in helping confirm where each laser exposure was located on the cornea. This camera also viewed the placement of the subject in real time using its live view feature, displayed on a large TV, to help ensure exposure sites did not overlap as the animal stage was oriented, and that the target was in focus.

### 2.2 Laser Exposures

With the subject in sternal recumbency on the stage and its eye prepped, it was then moved to within the vicinity of the exposure plane designated by the projected target. The animal was then finely maneuvered using the stage’s motors until the target came into focus on the cornea. Importantly, the focus of the target is synonymous with the location of the beam waist of the exposure laser and was used to measure the radiant exposure delivered. The camera observing the exposures was then adjusted for focus and centered on the eye. The camera’s live preview was projected on a large monitor for the staff to observe where the target was on the cornea. This allowed other personnel to identify any issues they observed as the animal was maneuvered on the stage to expose different areas of the cornea. The main issues to be avoided were exposure already placed in an area based on a map and identifying dry/rough patches of the cornea observed during baseline imaging.

When an adequate exposure location was identified, the eye was rinsed with normal saline and a 30-s countdown began, during which a pre-exposure image of the eye was acquired. After the 30 s had passed, the eye was exposed to the laser and a postexposure image was acquired. Regular rinsing of the eye began again, whereas the subject was maneuvered to find a new corneal exposure location. Regulating the time after the eye was last rinsed before an exposure was important for normalizing the volume of water coating the eye. As water absorption is the main attenuator of the laser at this wavelength, changes in water content can potentially affect the results of whether damage is caused.

Each eye was exposed 9 to 11 times. The images taken before and after an exposure were useful for identifying whether the eye moved during the laser exposure. Aside from limiting eye movement, sutures were also placed in an attempt to provide landmarks for identifying exposure locations; there was middling success in this regard. In the end, the most reliable method of identifying lesion locations was by referring to the exposure images and comparing the target positioning to them.

### 2.3 Lesion Identification

During postexposure examinations, a board-certified optometrist operated the slit lamp microscope, with and without fluorescein, to identify any alterations of the cornea or lens. Images were acquired of the entire cornea and of any alterations detected. Alterations detected by the slit lamp were also imaged using anterior segment OCT to acquire more information about depth. The size of identified lesions was estimated using the FOV and magnification of the imaging instruments. The lesions found during postexposure examinations were corroborated using images taken during the exposures to match lesions with the radiant exposure that created them, resulting in a final score.

### 2.4 Probit Analysis

Probit is a statistical method for analyzing dose-response data, which assumes the data has a log-normal probability distribution.^50^ For this study, utilizing Probit version 2.1.3 (1998), it found an ${\mathrm{ED}}_{50}$, or radiant exposure dose, that is expected to cause an injury for 50% of exposures, and the 95% confidence interval surrounding it (also called fiducial limits). These upper and lower limits represent the standard error associated with the ${\mathrm{ED}}_{50}$ threshold that would be commonly plotted as error bars on a bar graph. The beam spot size was measured daily and used to calculate the radiant exposure per set of exposures. Dose-response data from five rabbit eyes for a total of 50 data points were collected to determine the corneal MVL ${\mathrm{ED}}_{50}$ and were considered adequate when the slope produced was greater than 5, and the fiducial limits were less than half of the ${\mathrm{ED}}_{50}$.

### 2.5 Histology

Five excised, formalin-fixed globes were submitted for pathologic evaluation by a board-certified veterinary pathologist. On receipt, each globe was evaluated macroscopically for visible lesions and survey images were collected using an Evident SZX16 stereomicroscope with the Evident cellSens Entry software package. Each globe was then reoriented to a standard anatomic position, and the cornea was removed using a #11 scalpel blade at the corneal limbus. Maintaining the same anatomic orientation, the ventral surface of the cornea was transected ∼1 to 2 mm dorsal from the ventral aspect using a #62 scalpel blade to provide a flat surface for embedding and microtomy. The remaining intact edges of the corneal limbus were then marked using six different color tissue inks in a radial pattern to facilitate tissue navigation and exposure site identification during histopathological evaluation. The inked corneas were imaged again using the same SZX16 stereomicroscope and then processed in a tissue processor, embedded in paraffin, and serially sectioned at a thickness of 4.5  μm. Every 10th section throughout the entirety of the exposure region was collected for histopathology, stained with hematoxylin and eosin (H&E), and placed on a glass slide with a cover slip.

Due to the volume of slides produced from each cornea, whole slide images (WSIs) were produced from the H&E slides internally using an Evident SLIDEVIEW VS200 research slide scanner and reviewed and annotated in sequence using the QuPath v0.5.0 open software for bioimage analysis. Survey slide images were captured using QuPath, and high-quality images of identified lesions were captured using the Evident cellSens Entry imaging software. In addition, an Olympus BX43 light microscope was used as needed to complement the WSI analysis. The reading pathologist reviewed and scored all slides blinded to the energy levels. The results for each subject were then documented and logged as predominantly semiquantitative, ordinal data in a data matrix.

## 3 Results and Discussion

**Fig. 4:**
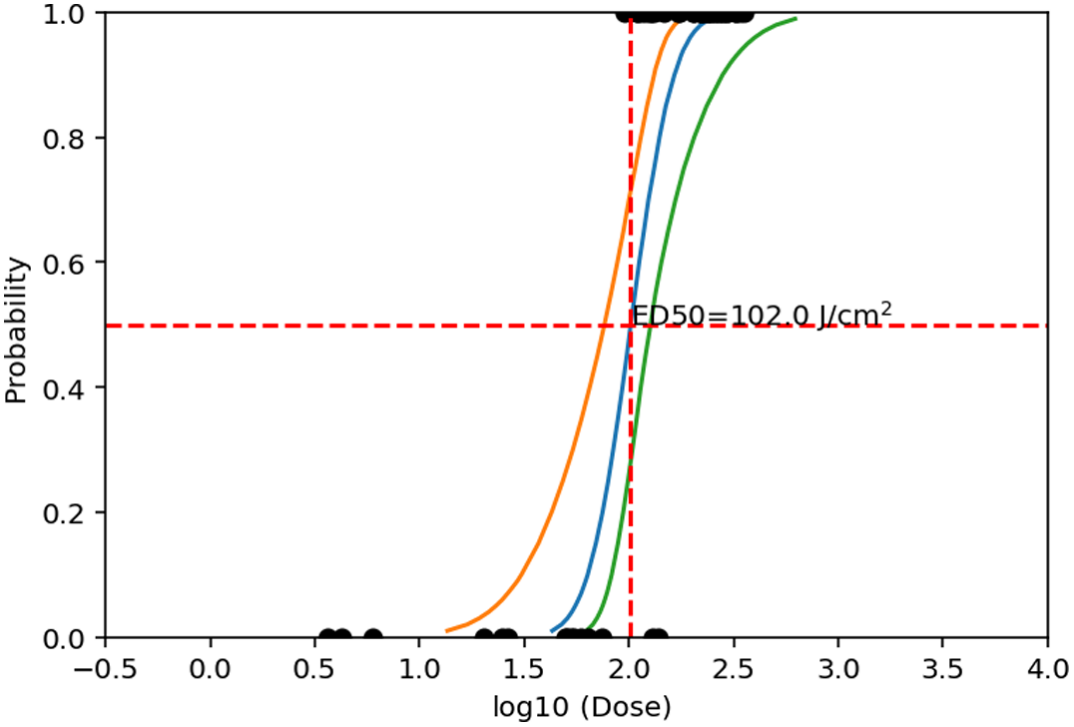
Plot depicting the Probit results of a peak radiant exposure of $102\;J/{\mathrm{cm}}^{2}$ causing a corneal minimally visible lesion (MVL) with a 50% probability from single-pulse exposures to a Q-switch laser with a central wavelength of 1645 nm. The beam diameter (69.47±2.42  μm at 1/e) was measured at the beam waist [exposure plane] via the knife-edge method every day before laser exposures. The orange and green lines depict the lower and upper limits of the 95% confidence interval peak radiant exposures of 76.4 and $127\;J/{\mathrm{cm}}^{2}$, respectively.

The maximum energy achieved at the exposure plane was 13.25±0.29  mJ. Based on this, the maximum peak radiant exposure per 77-ns pulse was $349.5\pm 14.0\;J/{\mathrm{cm}}^{2}$. Uncertainties of the radiant exposure measurement propagated from the delivered energy measurement and the knife edge measurement of beam diameter. This included uncertainties of the energy detector (2%) and meter (1%), the laser noise between pre- and postexposure calibrations for the energy delivered measurement (1.8%), and the micrometer used for the knife edge measurement (1%) leading to beam diameter uncertainty of 3.5%, energy delivered uncertainty of 2.2%, and radiant exposure uncertainty of 4.1%. Maximizing radiant exposure through the laser system was important for determining an ${\mathrm{ED}}_{50}$ using Probit analysis, which requires a range of data points below and above the threshold value to arrive at a statistically robust threshold. The energy ${\mathrm{ED}}_{50}$ value was 3.86±0.085  mJ. For a ${1/e}^{2}$ spot diameter of 98.25±3.4  μm (1/e diameter: 69.47±2.4  μm) at the exposure plane, this corresponds to a peak radiant exposure of $102\;J/{\mathrm{cm}}^{2}$. The upper and lower 95% confidence intervals of the radiant exposure ${\mathrm{ED}}_{50}$ were 127 and $76.4\;J/{\mathrm{cm}}^{2}$, respectively. The Probit slope was 6.19 at the ${\mathrm{ED}}_{50}$ value (see Fig. 4). By contrast, the average radiant exposure ${\mathrm{ED}}_{50}$ over a 1-mm-diameter limiting aperture as per the ANSI Z.136 convention was $0.49\;J/{\mathrm{cm}}^{2}$.

This ${\mathrm{ED}}_{50}$ threshold was notably larger than the total delivered peak radiant exposure window (39.5 to $53.7\;J/{\mathrm{cm}}^{2}$) found in the pilot study in swine using $3.58\;J/{\mathrm{cm}}^{2}$ per pulse exposures. The discrepancy is due, in part, to the difference in the beam spot size, number of pulses, and the influence the beam diameter has on thermal transport, as well as a change in species tissue (porcine versus rabbit). The current focused beam diameter at the focal plane (rabbit) cornea was ∼10 times smaller than the pilot study’s collimated beam diameter (swine). Larger spot sizes require less energy to increase the temperature of the exposed tissue, whereas smaller spot sizes allow heat to diffuse away from the exposure site more rapidly, stunting heat buildup. This hypothesis was tested using the Scalable Effects Simulation Environment (SESE, version 2.8.0) model developed by Nanohmics, Inc. (Austin, TX), in collaboration with the AFRL. SESE is a physics-based software package designed to solve biophotonic-related problems.^51^ The simulation used the same laser parameters (e.g., wavelength, pulse energy, and pulse duration) except for beam diameter. These different spot sizes were used for exposures of a slab of material with similar thickness and optical properties as the cornea. The results showed an increase in tissue temperature of 2°C after two pulses. Inputting the experimental parameters into SESE necessitates a two-pulse train rather than a single pulse, which would result in a CW simulation. This increase in temperature for a 1-mm beam diameter exposure compared with a 100-μm beam diameter exposure suggests a higher likelihood of damage occurring for the larger beam (confirmed with pilot study results). Additional work using this SESE model is being investigated with *in vitro* tissue results using the same laser as this study.

**Fig. 5:**
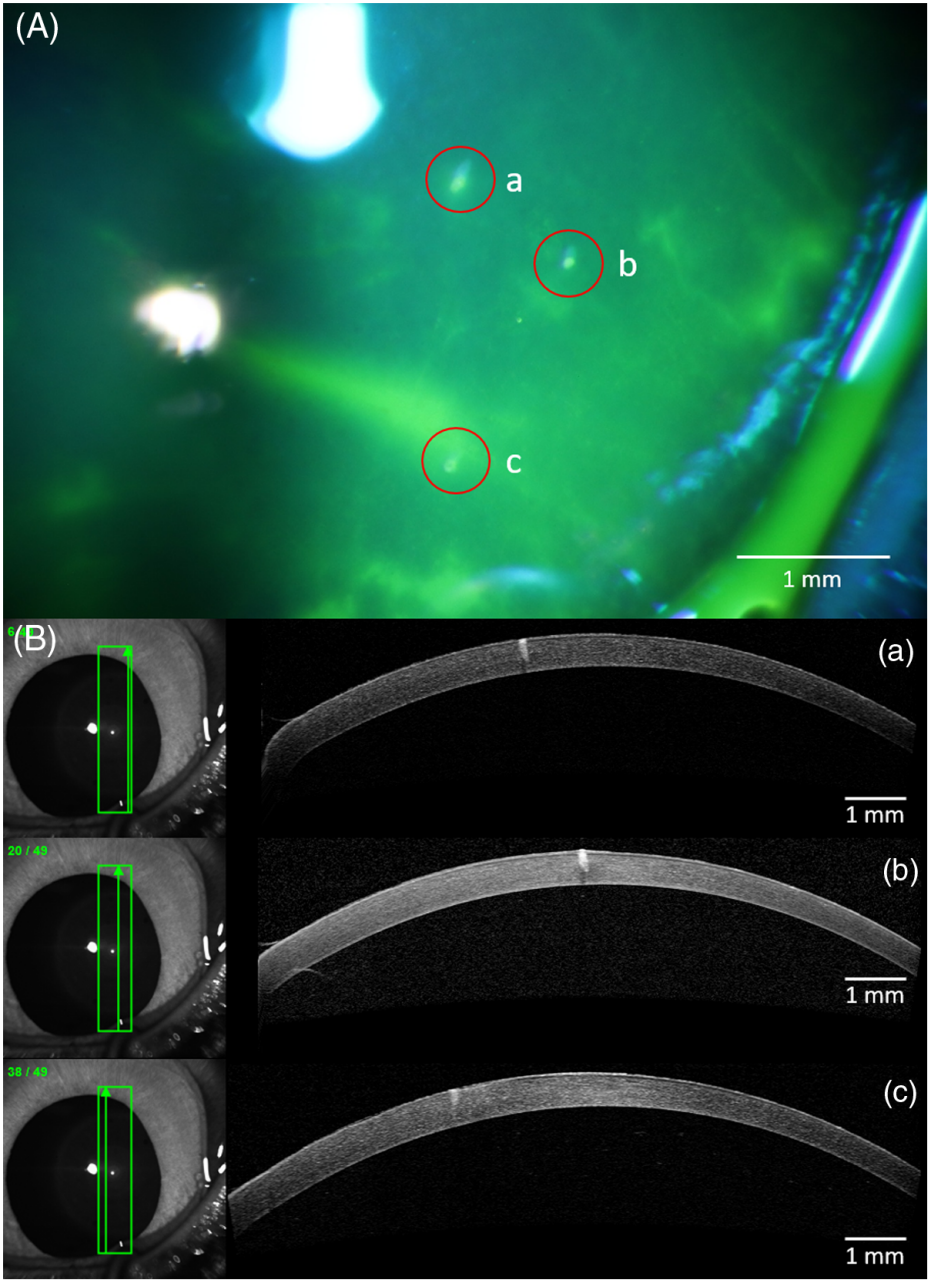
Examples of corneal lesions caused by a Q-switched laser at 1645 nm. The top panel (A) was acquired using slit lamp microscopy with fluorescein staining highlighting three exposures: the images in the bottom panel (B) correspond to OCT B-scans taken at the three sites exposed to different peak radiant exposures: (a) $267\;J/{\mathrm{cm}}^{2}$, (b) $218.7\;J/{\mathrm{cm}}^{2}$, and (c) $199\;J/{\mathrm{cm}}^{2}$. The eye in the slit lamp image is rotated [rolled] clockwise from its position in the OCT images. Identifying exposure locations could be complicated by drastic rolling of the eyes changing exposure positioning, particularly when the subject’s eyes were closed during transportation, when their positioning is not being actively tracked by observers.

Some examples of corneal lesions caused by exposure to a single, ns pulse at 1645 nm are shown in Fig. 5. Fluorescein was applied to the cornea to help highlight lesions. Of note, areas of dryness on the cornea related to anesthesia effects were also highlighted by fluorescein. Dry areas were intentionally avoided for data collection because they might change damage thresholds (different water content) and could hinder lesion identification. Anterior segment OCT images assisted in the identification of lesions caused by laser exposures. For the exposure conditions used in this illustration, the OCT images showed laser lesions extending into the stroma, whereas superficial epithelial disruptions, such as those caused by dryness, did not. The results of Fig. 5 emphasize the need to acquire OCT images in combination with slit lamp microscopy for confirmation of damage depth. The greatest transverse resolution for B-scans by the Heidelberg Spectralis was 16  μm, so there is no assurance that a given cross-section is at the center of a lesion. This uncertainty introduces possible errors in measuring lesion diameter using OCT.

**Fig. 6:**
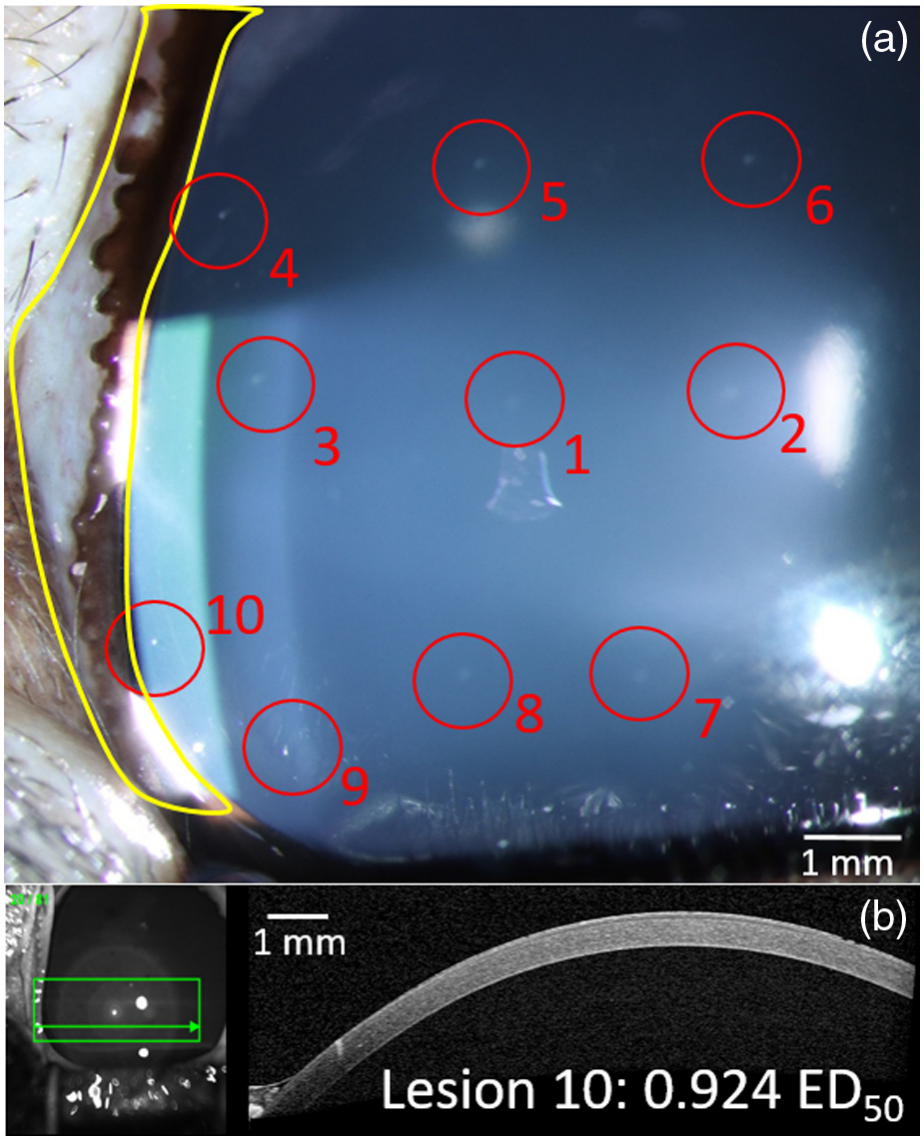
Corneal lesions imaged by slit lamp biomicroscopy 1 h post-laser exposure are shown in panel (a). Lesions are encircled in red and numbered according to the order of exposure made. Laser exposure sites on the cornea were identified in this manner before scanning the exposures with OCT. The eye was then taken for histological analysis of the injuries. Histological images of a number of these lesions are depicted in Fig. 8. Lesion 10 was generated by a peak radiant exposure of 0.924 of the ${\mathrm{ED}}_{50}$ value ($94.2\;J/{\mathrm{cm}}^{2}$), and an OCT B-scan corresponding to this is shown in panel (b). The subject’s nictitating membrane eyelid is marked in yellow and caused difficulties in identifying lesions proximal to the eye due to its movement covering them at times such as with lesion 10.

An example of a lesion caused by a radiant exposure of 92% of the determined ${\mathrm{ED}}_{50}$ threshold is shown in Fig. 6 (lesion 10). As an aside, the subject’s tertiary eyelid (nictitating membrane) can be seen next to the left of the lesion as the white membrane with an undulant pigmented pattern encircled by a yellow line. Due to the rabbit’s ultrawide field of vision and eye positioning on the side of the head, the ability for their eyes to roll to extreme distances in various directions required some externally applied manipulations. At times, due to this rolling, the lesions would be observed near the edges of the field of view or be nearly covered by the nictitating membrane (examples in Fig. 6).

**Fig. 7:**
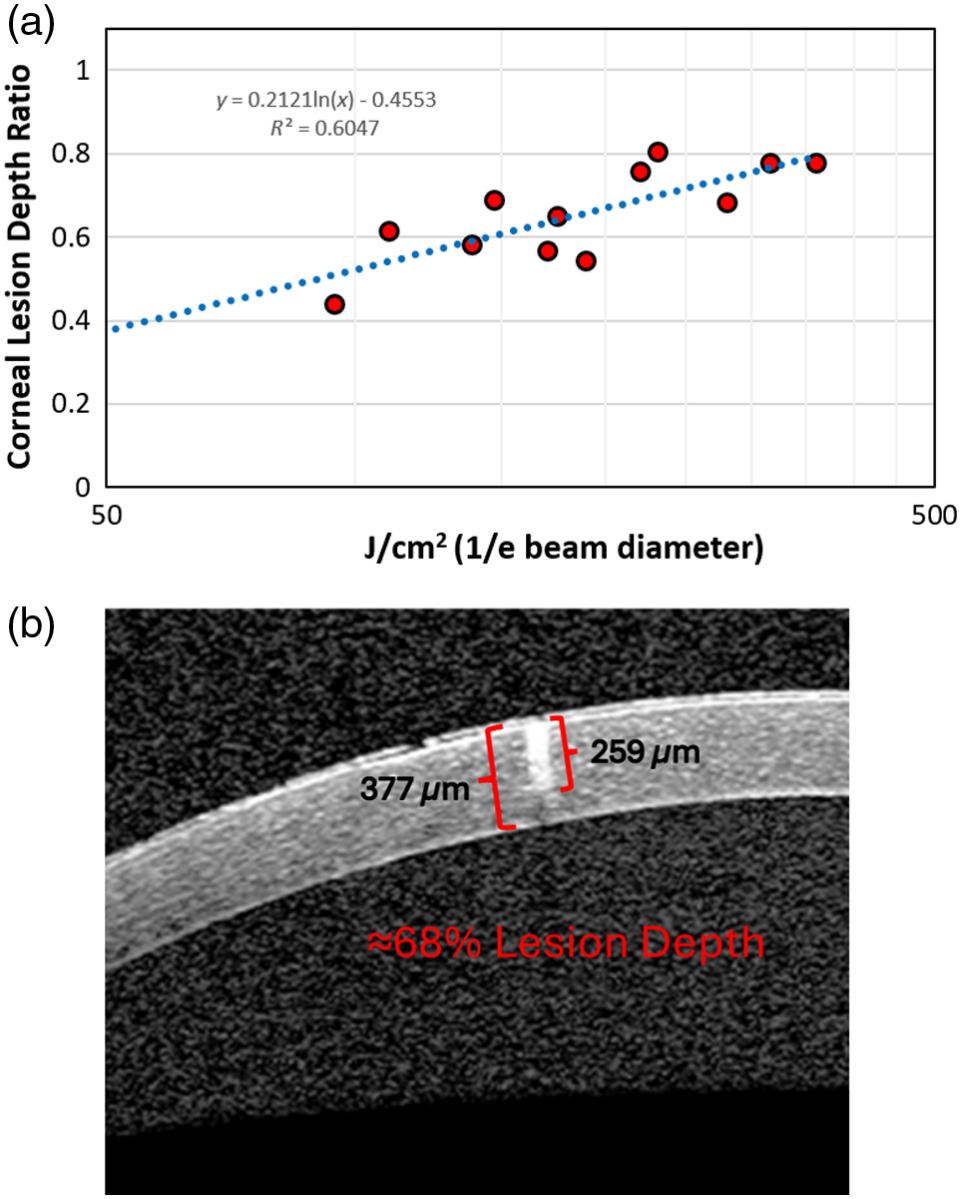
Correlation of the depth of corneal lesion with radiant exposure dose. (a) Plot of lesion depth as a percentage of full corneal thickness and laser radiant exposure showing a positive correlation. (b) An example of measuring the corneal lesion depth ratio using an OCT image. Brackets identify the corneal and lesion thickness where the lesion extended ∼68% through the cornea.

As a measure of damage severity, the ratio of lesion depth to total corneal thickness at an adjacent location was used (see Fig. 7). A ratio of 1 indicates that the lesion extended the full thickness of the cornea. The mean full corneal thickness at lesion locations was 381  μm (range 323 to 417  μm). Figure 7(a) shows the relationship between the lesion depth ratio to laser radiant exposure delivered. The relationship between damage depth and energy delivered appears to be logarithmic. This suggests that as the radiant exposure is increased, additional energy is needed to develop deeper and deeper lesions. For example, by doubling the energy, the depth of damage would be expected to only increase by 123%. The correlation between lesion depth and energy level was strong, with a coefficient of determination ($R^{2}$) of 0.7455. This value indicates that, according to this model, any variance of depth at a determined energy would be expected to be within 39% of the trend line.

Figure 7(b) highlights an example measurement from an anterior segment OCT B-scan image. Red brackets on the image indicate where depth was measured. For this lesion, the damage depth was measured at 259  μm and the full thickness at 377  μm. This depicts a 68% depth lesion (0.68 depth ratio).

**Fig. 8:**
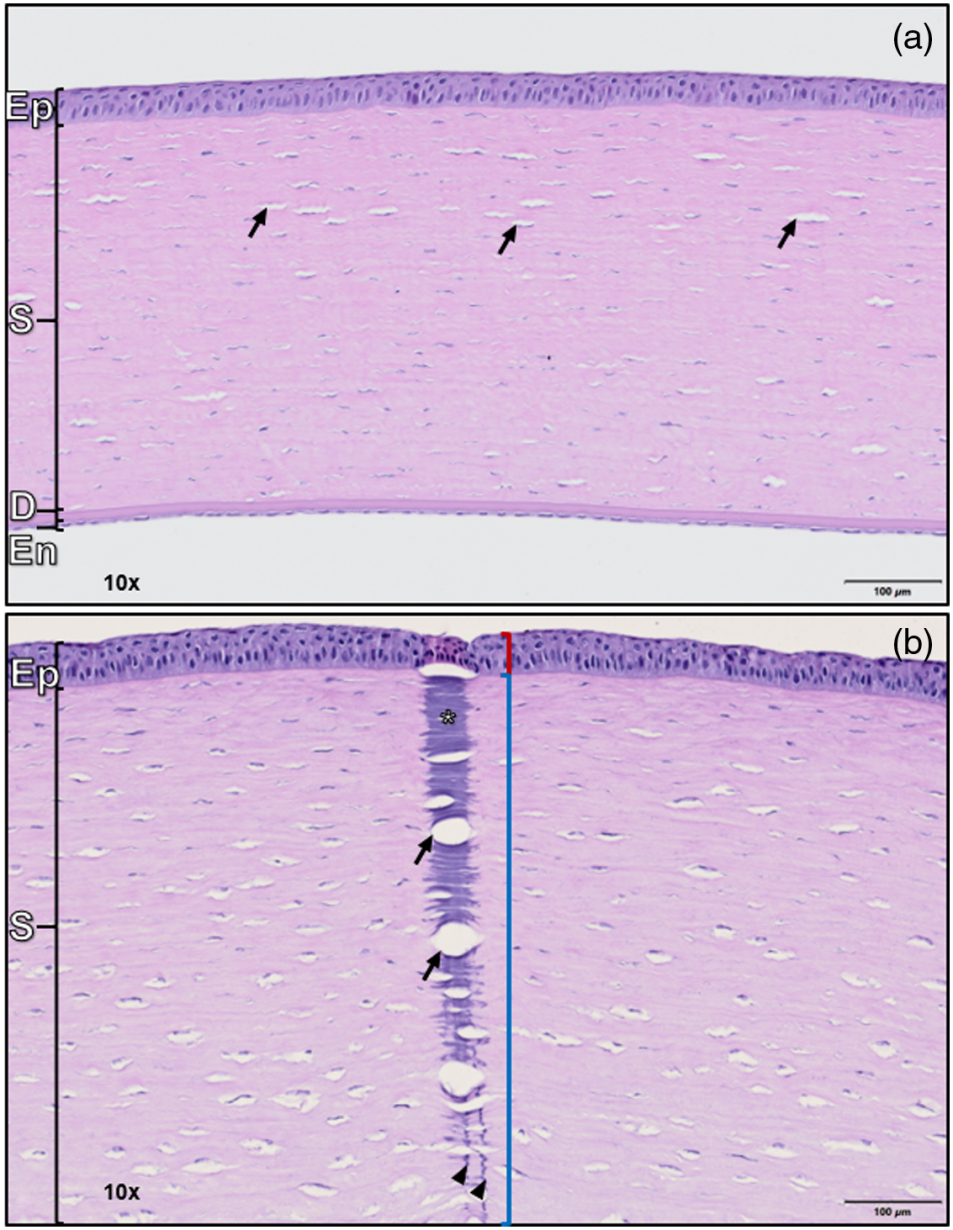
Example of (a) normal and (b) damaged rabbit cornea. The scale bar for both images is 100  μm. The layers are organized from outer (top) to inner (bottom): corneal epithelium (Ep), corneal stroma (S), Descemet’s membrane (D), and corneal endothelium (En). The black arrows point to examples of stroma clefts under normal and damaged tissue conditions. H&E, WSI scanned with an Evident VS200 slide scanner at 40× resolution.

A representative example of a normal and damaged rabbit cornea is shown in Fig. 8. The corneal epithelium located at the top (outermost) layer protects the cornea from external environmental factors and prevents fluid transfer into the stroma. The corneal stroma is composed of predominantly collagen fibers with stromal fibroblasts (keratocytes) and variable numbers of clear spaces (clefts; arrows), which vary in size and number by species. Descemet’s membrane is a durable membrane that is situated beneath the stroma, and the corneal endothelium is the innermost layer that forms a watertight seal to prevent leakage of aqueous humor into the corneal stroma. The inner and outermost layers prevent an excess of water from entering the stroma, which makes up the bulk of corneal tissue.

After exposure to the laser, histologically identifiable tissue damage extends from the corneal surface down through the corneal epithelium and into the underlying corneal stroma [see Fig. 8(B)]. The damaged corneal epithelium (red bracket) is composed of shrunken corneal keratinocytes with hypereosinophilic cytoplasm and pyknotic nuclei with loss of attachment to the underlying basement membrane (necrosis). The damaged corneal stroma (blue bracket) consists of basophilic denatured collagen fibers (asterisk) with interspersed expanded stromal clefts (arrows) and loss of identifiable stromal fibroblasts (keratocytes). There is a sharply demarcated, conical to cylindrical-shaped profile and basophilic vertical streaking extending into the deep end of the stroma (arrowheads).

The morphology of the damage caused by the single pulse exposures from the Q-switched laser is not what is expected from contact burns or even CW lasers. Contact burns typically concentrate at the surface with collagen damage radiating downward through the stroma in a uniform and diffusive shape. The epithelial damage and initial conical damage profile in the stroma are similar to thermal damage produced by CW lasers in the cornea. However, the expanded stromal clefts and streaks extending deeper into the cornea are not typical for CW laser injuries. Rather, the shape of the cellular damage highlighted by H&E staining suggests mechanical and thermal modes of damage working in tandem. It has been shown that pressure applied to proteins can not only cause denaturation but can also lower the temperature threshold for denaturation.^52^^–^^54^ It is hypothesized that the nanosecond laser pulse generated a stress wave within the tissue that may have caused a reduction in the heat required to denature the tissue, resulting in a larger volume of damage than expected. In addition, the stress wave itself may be responsible for branching streaks of stromal damage caused by photomechanical stress as the laser propagated into the tissue more efficiently in certain tracts of stroma rather than propagating uniformly through the tissue. No blood vessels are present in the corneal stroma, refuting the idea that heat may be propagating through a blood vessel or hair follicle, which can occur in the skin.

**Fig. 9:**
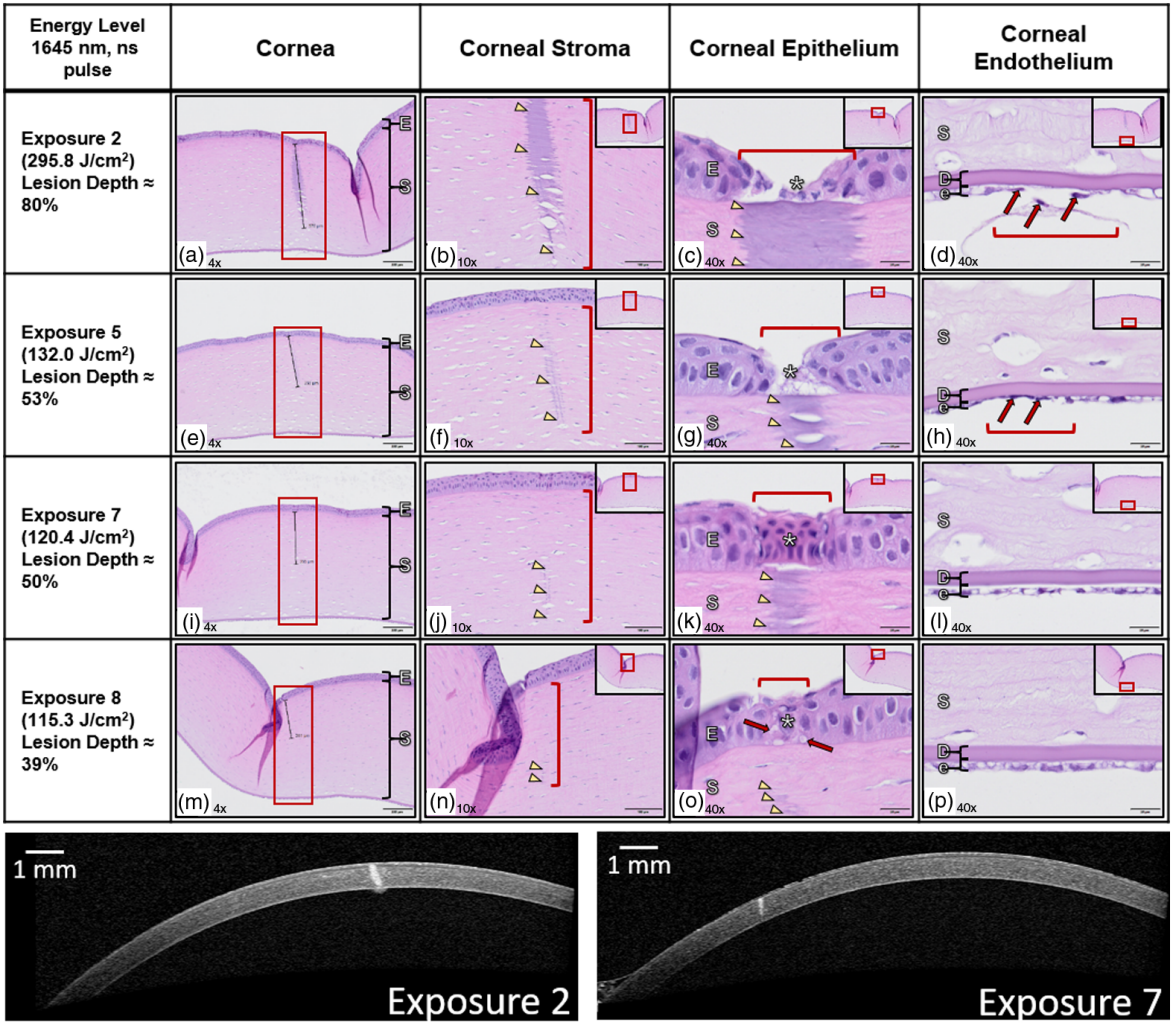
Representative cornea example of histological and OCT analysis. Scale bars are at the bottom right of each panel. Panels a, e, i, m (scalebar: 200  μm): Histologically identifiable tissue damage extended from the corneal surface (depending on slide section) down through the epithelium, into the corneal stroma, and at higher energies, affected the underlying corneal endothelium (red boxes). Panels b, f, j, n (scalebar: 100  μm): Corneal stromal damage consisted of basophilic denatured collagen arranged in obliqued linear profiles (arrowheads, red brackets). Panels c, g, k, o (scalebar: 20  μm): The damaged corneal epithelium (*, red brackets) was ablated at higher energies with exposure of the underlying, thermally damaged corneal stroma (arrowheads), and at lower energies, was either necrotic with shrunken, hypereosinophilic epithelial cells with elongated nuclei, or degenerate with variably vacuolated cytoplasm (red arrows). Panels d, h, l, p (scalebar: 20  μm): The underlying corneal endothelium was damaged in the higher energy exposures only (d, h, red brackets), characterized by shrunken cells with pyknotic nuclei (arrows). Corneal epithelium (E), corneal stroma (S), Descemet’s membrane (D), and corneal endothelium (e). H&E, all WSIs scanned with an Evident VS200 slide scanner at 40× resolution.

Additional H&E-stained images of corneal laser exposures are shown in Fig. 9, with anterior segment OCT images corresponding to two of the exposures for comparison. The images were taken from the same corneal tissue depicted in Fig. 6. The lesions represent a range of peak radiant exposure doses greater than the measured threshold ${\mathrm{ED}}_{50}$ of $102\;J/{\mathrm{cm}}^{2}$, listed in the first column from greatest to lowest. The lesions observed postexposure were identified and linked to the corresponding radiant exposure dosage during the histological analysis of the corneas based on their positioning, and the ratio of the lesion depth to corneal thickness is listed per exposure. The pathologist was blind to exposure dosage. The injury is marked by a red rectangle in the second column [see Figs. 9(a), 9(e), 9(i), 9(m)]. The depth of tissue damage is marked with a black measuring bar, with Fig. 9(a) being 570  μm deep. The depth of the damage increased as a function of radiant exposure dosage through the epithelium and partway through the corneal stroma. This tissue damage should not be confused with the crease and fold artifacts that formed in the cornea as a part of the histology preparation process.

A closer look at the corneal stromal damage is shown in the third column [see Figs. 9(b), 9(f), 9(j), 9(n)] as basophilic (darker-stained) denatured collagen, identified by the arrowheads and red brackets. The shape of the damaged stromal tissue is of interest because it differed from what is normally expected from a purely thermal mechanism. An exposure from CW directed energy is expected to generate the largest extent of damage in the most anterior aspect of the cornea, gradually decreasing in damage diameter posteriorly as heat is diffused. Figures 9(a) and 9(b) show the largest extent of damage (width) occurring within the basophilic streak of damaged stromal tissue, which appears as an oblong to cylindrical-shaped discoloration, again suggesting that there may be more than just a thermal damage mechanism at play similar to Fig. 8(b).

Tissue damage morphology corresponded to the different kinds of tissue that make up each corneal layer. Damage to the epithelial layer depended on exposure energy level [see Figs. 9(c), 9(g), 9(k), 9(o)]. Higher energies ablated the epithelium, leaving the denatured stroma exposed underneath. Lower energies left the epithelium necrotic, with shrunken epithelial cells and elongated nuclei, or degenerate, with vacuolated cytoplasm. Beneath the corneal stroma and Descemet’s membrane, the corneal endothelium was damaged at higher energies, resulting in shrunken cells and pyknotic nuclei [see Figs. 9(d) and 9(h)]. Lower energy exposures appeared to leave the endothelium unaffected. Descemet’s membrane was not noticeably altered histologically on H&E staining for any of the laser exposures, even when the endothelium underneath was damaged. This could be in part due to the lower elastic modulus reported for the Descemet’s membrane as compared with the stroma.^55^ Photothermal energy and photomechanical stress caused by the laser exposures may not be enough to visibly alter the deepest portion of the stroma or Descemet’s membrane. However, there appears to be enough energy transmitted through the stroma to disrupt the single-cell endothelial layer.

An example of anterior segment OCT images of the cornea for two of the exposures is shown in the final row of Fig. 9. The change in optical properties of the damaged tissue is manifested as a white streak through the cornea. The difference in the size of the injury between exposures 2 ($295.8\pm 12.0\;J/{\mathrm{cm}}^{2}$) and 7 ($120.4\pm 4.9\;J/{\mathrm{cm}}^{2}$) can be discerned by comparing their widths and depths. Plotting the ratio of lesion depth to total corneal thickness against the radiant exposure dose as was done in Fig. 7(a) gave an $R^{2}$ value of 0.9201 compared with 0.7455 found with the OCT example. The improvement was likely due to the increased resolution of the microscope images. The basophilic streaks seen at the deeper end of the stroma in the histology images are not obviously discernible in the OCT images. These streaks are included in the calculation of the corneal lesion depth ratio using the histology data.

## 4 Conclusion

This study presents the first set of experimental data assessing Q-switched laser corneal injury at a central wavelength of 1645 nm. The current ANSI Z136.1 point-source MPE is $1\;J/{\mathrm{cm}}^{2}$ for a single pulse at 1645 nm within the ns pulse duration regime. Using the suggested 1-mm limiting aperture to calculate the average radiant exposure, the damage threshold reported here resulted in an ${\mathrm{ED}}_{50}$ of $0.49\;J/{\mathrm{cm}}^{2}$. This value is half of the current MPE limit listed in the ANSI Z136.1, suggesting that the current MPE is too permissive. At this MPE, twice as much radiant exposure would be permitted per exposure than what produced damage in this study. At this current recommended MPE, damage will occur.

However, calculating the peak radiant exposure threshold using the experimental spot size resulted in $102\;J/{\mathrm{cm}}^{2}$. The 200-fold difference between peak and average radiant exposure ${\mathrm{ED}}_{50}$ values necessitates a discussion on how to interpret the damage threshold in cases where the beam diameter is smaller than the 1-mm limiting aperture.

In general, the safety margin for consideration in the ANSI Z136.1 standard aims for a ratio of ${\mathrm{ED}}_{50}/\mathrm{MPE}=10$. For an MPE of $1\;J/{\mathrm{cm}}^{2}$, the ${\mathrm{ED}}_{50}$ values based on peak ($102\;J/{\mathrm{cm}}^{2}$) and average ($0.49\;J/{\mathrm{cm}}^{2}$) radiant exposures have ratios of ∼100 and 0.5, respectively. The former ratio indicates an overly restrictive MPE (${\mathrm{ED}}_{50}$ is 100× higher than MPE), whereas the latter indicates an overly permissive MPE. A possible adjustment to the safety standard, to account for beam diameters less than the limiting aperture while considering beam diameter, is to express the ${\mathrm{ED}}_{50}$ as the average radiant exposure over the ${1/e}^{2}$ diameter of the beam. This is 0.5 times the peak radiant exposure, resulting in an ${\mathrm{ED}}_{50}$ of $51.2\;J/{\mathrm{cm}}^{2}$ for the results presented here.

The histological analysis of resulting corneal damage in this study suggests that both photothermal and photomechanical mechanisms play a role in causing injury. The morphology of stromal damage shown by H&E staining is incongruent with what is typically expected with purely thermal injuries. This lends credence to the idea that the mechanism of injury caused by pulsed lasers begins to shift from predominantly thermal with CW lasers toward photomechanical at nanosecond and shorter pulse durations.

Anterior segment OCT images complemented the slit lamp microscope images for lesion identification. This was especially useful when the cornea was dry and/or rough during baseline imaging. Applying fluorescein to the cornea during slit lamp microscopy highlighted both the lesions caused by the laser and areas of roughness. OCT can help differentiate between the two features, because corneal dryness and surface roughness are superficial, whereas laser lesions extend deep into the cornea at higher radiant exposure levels.

The rabbit corneal Probit ${\mathrm{ED}}_{50}$ threshold radiant exposure of $102\;J/{\mathrm{cm}}^{2}$ was roughly double the total delivered radiant exposure for corneal injury found in the pilot study. Although the same laser was used for both experiments, the order of magnitude difference in beam size made a tangible effect on the tissue temperature rise that contributed to injury. By not having sufficient laser energy in the pilot study to produce damage with a single pulse at the larger beam diameter and the difference in species, the earlier data are not directly comparable to our current data. Although the rabbit model has been utilized for numerous ocular studies, there remain structural and cellular differences of the tissue when compared with similar models (human, nonhuman primate, or porcine). This is especially true for laser–tissue interaction experiments. Further research is needed to address data gaps related to those differences in models (ocular quality, tissue composition, tissue elastic properties, etc.). Future studies are planned to examine these results in the context of other wavelength experiments and the predicted outcomes more broadly through physics-level modeling and simulation analyses. Extending the analysis will inform considerations of exposure limit standards across the NIR parameter space.

## Data Availability

The data that support the findings of this study are available from the corresponding author upon reasonable request.

## Distribution Statement

Approved for public release; distribution unlimited. PA Case No: AFRL-2025-499. The opinions expressed in this document, electronic or otherwise, are solely those of the author(s). They do not represent an endorsement by or the views of the United States Air Force, the Department of Defense, or the United States Government.
